# Clinical observation of the treatment of refractory cancer pain with cancer pain information platform and IDDS under home analgesia mode: A retrospective study

**DOI:** 10.1097/MD.0000000000038765

**Published:** 2024-07-05

**Authors:** Yongyong Ding, Hong Deng, Jie Peng

**Affiliations:** a Department of Pain, The First People’s Hospital of Zunyi (The Third Affiliated Hospital of Zunyi Medical University), Zunyi, China; b Department of Endocrinology, The First People’s Hospital of Zunyi (The Third Affiliated Hospital of Zunyi Medical University), Zunyi, China.

**Keywords:** cancer treatment, home analgesia, information platform, intrathecal drug delivery systems, refractory cancer pain, semi-implantable

## Abstract

To evaluate the effectiveness and safety of a cancer pain information platform combined with semi-implantable intrathecal drug delivery systems among the patients with refractory cancer pain under a “home analgesia” model. This was a retrospective study. A total of 49 patients underwent semi-implantable intrathecal drug delivery systems with patient-controlled analgesia in conjunction with the establishment of a cancer pain information platform. Numeric rating scales (NRS), Bruggrmann comfort scale (BCS), high-quality sleep duration, and opioid-related adverse effects were recorded at various time points and analyzed: the day on admission (T0), the day of discharge (T1), 30 days post-discharge (T2), 60 days post-discharge (T3), 90 days post-discharge (T4), 120 days post-discharge (T5), 150 days post-discharge (T6), 180 days post-discharge (T7), and the day before death (T8). Compared with T0, NRS significantly decreased and BCS significantly increased at T1 to T8 time points (*P* < .05). However, NRS and BCS did not show differences at T1 to T8 time points (*P* > .05). The duration of high-quality sleep was significantly extended, and the incidence of opioid-related adverse effects was significantly reduced. Postoperative complications included 1 case of cerebrospinal fluid leakage, 3 cases of infection at the butterfly needle insertion site, 6 cases of hospital readmission for equipment malfunction, and no cases of respiratory depression. Eleven patients continued standardized antitreatment after IDDS surgery. The mean survival time for all patients was 135.51 ± 102.69 days, and the survival rate at T7 was 30.61%. The cancer pain information platform combined with semi-implantable IDDS is beneficial for the pain management of refractory cancer patients under the “home analgesia” model, improving their quality of life.

## 1. Introduction

Cancer pain, especially refractory cancer pain, is a common symptom among cancer patients.^[1]^ It brings immense suffering to patients due to its long duration and severe intensity. Besides affecting their daily life and work, refractory cancer pain can also lead to psychological issues such as anxiety and depression, further exacerbating the pain.^[2]^ For patients with refractory cancer pain, multiple aspects need to be addressed, including pain treatment, psychological support, mitigation of treatment side effects, improvement of social support, and popularize knowledge and understanding of pain, to comprehensively improve their quality of life.^[3]^ However, among the numerous factors that impact quality of life, the management of pain intensity is particularly crucial. However, cancer pain management faces numerous challenges, such as inaccurate pain assessment, inappropriate drug treatment, insufficient patient education and knowledge and popularize the lack of cancer pain cognition to patients. These challenges result in many patients not receiving effective pain control, adversely impacting their quality of life. Besides aggressive antitumor treatment, intrathecal opioid infusion is a safe and effective method for treating refractory cancer pain.^[3–8]^

The “home analgesia” model is a novel pain management approach that extends treatment and care into the home environment. This model offers advantages in terms of convenience, personalization, reducing medical costs, improving quality of life, and facilitating multidisciplinary collaboration.^[9]^

The “home analgesia” concept circumvents the inconvenience associated with conventional hospital-based treatment, diminishes medical expenses, and enhances the quality of life for patients. Moreover, the multidisciplinary collaboration model offers a wide range of therapy services and enhances treatment results. The “home analgesia” mode employs a holistic approach that combines many treatment approaches, such as medical treatment, physical therapy, psychiatric therapy, home care, community services, pain education, and knowledge dissemination. Medication is the primary approach for alleviating pain, while physical and psychological therapy can offer further comfort. Home care and community programmes offer essential daily care and medical assistance for patients. Furthermore, the provision of instruction on pain and the transmission of knowledge can enhance the cognitive capacity of patients and their families, enabling them to accurately comprehend and manage pain-related matters.^[10]^ The difficulty of “home analgesia” patients lies in the inability to dynamically monitor the patient’s pain and timely deal with emergencies in the process of analgesia, which makes the effect of analgesia unsatisfactory.

Therefore, our hospital establishes an Information Platform for Cancer Pain Management. The patient carries out self-evaluation on the information platform according to his actual situation, and the physician carries out timely assessment according to the content of the patient’s self-evaluation, takes the initiative to guide the patient or his family to carry out preliminary treatment. This study compiled the pain-relieving information of 49 patients with difficult-to-treat cancer-related pain who received therapy with a partially implanted intrathecal drug delivery system (IDDS) under the Information Platform for Cancer Pain Management in an at-home pain management mode.

## 2. Methods

### 2.1. General information

This retrospective analysis comprised 49 patients who received semi-implantable IDDS for refractory cancer pain and were administered home analgesia between January 2019 and September 2022 at our institution. All patients had a confirmed diagnosis of primary malignancy (including tumor recurrence after surgery), metastatic involvement of multiple organs at admission, and had lost the opportunity for surgical resection. They had received standardized antitumor treatment, including chemotherapy, radiotherapy, and targeted therapy, as well as “3-step analgesia” for cancer pain, but still experienced moderate to severe pain or intolerable adverse reactions to analgesic medications that prevented continuous treatment. The patients and their families requested to continue or discontinue antitumor treatment under adequate analgesia, with the primary goal of improving quality of life at the end stage.

During home analgesia, when the patient-controlled analgesia (PCA) pump’s drug reservoir was empty, patients visited the pain clinic to replace the reservoir and butterfly needle. The drug concentration and infusion system parameters were re based on the patient’s pain intensity and PCA frequency over the previous 3 days. Simultaneously, the skin at the butterfly needle insertion site was disinfected.

*Inclusion criteria: R*efractory cancer pain diagnosis according to criteria^[1]^: moderate to severe persistent cancer pain with a numeric rating scales (NRS) ≥ 4, with or without breakthrough pain ≥ 3 times per day; adherence to guidelines for the management of cancer pain, such as the NCCN Clinical Practice Guidelines for Adult Cancer Pain and the Cancer Pain Diagnosis and Treatment Specification (2018 version), but refractory to opioid analgesics alone or in combination with adjunctive medications after 1 week of treatment (NRS ≥ 7) or after 2 weeks of treatment (NRS ≥ 4), or due to intolerable adverse reactions.^[6,7]^

Multidisciplinary evaluation estimating a survival time of <3 months. *Exclusion criteria:* severe heart, lung, or liver failure; systemic severe infections that were not controlled; mental illness or patients who refuse implantation of the semi-implantable IDDS; surgical site infection or coagulation dysfunction that had not been corrected; regular WHO “3-step analgesia” treatment with an NRS ≤ 4 and no severe adverse drug reactions; evaluation estimating a survival time of <1 month and the patient is unaware of their condition.

This study was approved by the ethics committee of The First People’s Hospital of Zunyi (The Third Affiliated Hospital of Zunyi Medical University) (No. [2021]-1-62) and written informed consents were obtained from all patients and their families.

### 2.2. Intravenous drip of morphine hydrochloride

All patients received intravenous drip infusion of 4 mg morphine hydrochloride (1 mL:10 mg, produced by NORTHEAST PHARM, Shenyang First Pharmaceutical Co., LTD, Shenyang, China. National Drug Code: H21022436; Narcotic drugs and psychotropic substances production lot number: TD2010-0006) in 100 mL of 0.9% normal saline using a PCA device. The infusion rate was adjusted to achieve a target NRS ≤ 3 and breakthrough pain ≤ 3 times per day, which was considered suitable for implantation of the IDDS. The mean preimplantation oral morphine dose was 378.98 ± 161.12 mg per person.

### 2.3. Implantation of the semi-implantable IDDS

The implantation procedure was completed in each patient under local anesthesia in about 30 minutes. The patients were positioned in a lateral decubitus position. The target was located at the midpoint of the L2 to L3 intervertebral space. A paramedian approach was used, with the entry point selected at the ventral aspect of the L4 vertebral body, close to the medial border of the left posterior superior iliac spine. Local anesthesia was achieved using 20 mL of 1% lidocaine hydrochloride injected into the subcutaneous tissue. An 18-G Tuohy needle was placed and progressed until the observation of cerebrospinal fluid (CSF) flow. Subsequently, the 20G intrathecal catheter was inserted and moved toward the target segment until it reached the desired place.^[8]^ The catheter location was verified by the presence of CSF, or CSF flowing through the catheter and the extraction of CSF from the distal end. Subsequently, the needle was removed from the catheter, and the catheter was securely attached to the fascia using sutures. A 3 to 5 cm cut was created in the middle, and the tissue beneath the skin was carefully separated to form a tunnel for the catheter. The catheter’s distal end was linked to an implanted port device, which was put through a little incision located to the side of the anterior superior iliac spine. The implanted port device and the intrathecal catheter were securely stitched to the fascia. Subsequently, the PCA device was linked to the implanted port device, and the infusion rate and bolus dose were configured based on the requirements of the patient. Set the pump speed ≥ 0.1 mL/h, PCA lock time for 30 minutes, and each single additional dose of 1 to 2 mL. The opioids were completely discontinued after intrathecal analgesia. The patients were educated on the proper utilization of the PCA device and underwent regular assessments to verify their attainment of effective pain relief with minimum adverse reactions. After the completion of wound healing and the patient’s discharge from the hospital, regular follow-up appointments were scheduled to check their ongoing recovery.

The implanted port device (ZS2 series) was provided by Suzhou Linhua Medical Equipment Co. Ltd (National Instrument No: 201831417172; Suzhou, China).

### 2.4. Development of an information platform for cancer pain management

Patients, family members, and healthcare providers were trained in standardized pain assessment, information system operation, and troubleshooting. A professional information software program was used to establish an information evaluation platform jointly managed by the attending physician and the patient. The platform included evaluation components for pain intensity and nature, breakthrough pain, sleep, mental status, appetite, and adverse drug reactions. Patients self-assessed their status on the platform based on their actual conditions, and doctors evaluated their status based on the self-assessment results and provided guidance on initial treatment or referral to the hospital when necessary. The platform also allowed for recording of NRS, Bruggrmann comfort scale (BCS) scores, sleep time, nausea, and vomiting, diet, respiratory depression, adverse event handling through the information platform, and hospital readmission due to equipment malfunction or other reasons.

### 2.5. Evaluation Indicators

The definitions for different timepoints are as follows: The day on admission (T0), the day of discharge (T1), 30 days post-discharge (T2), 60 days post-discharge (T3), 90 days post-discharge (T4), 120 days post-discharge (T5), 150 days post-discharge (T6), 180 days post-discharge (T7), and the day before death (T8).

(1) *Pain intensity evaluation:* The digital NRS is used to evaluate the pain intensity of patients at T0 to T8 time points. The score range is from 0 (no pain) to 10 (severe pain).^[11]^ Lower scores indicate more favorable treatment outcomes.

- 0: No pain.
- 1 to 3: Mild pain, does not affect sleep and daily activities.
- 4 to 6: Moderate pain, affects sleep but does not affect daily activities.
- 7 to 9: Severe pain, significantly affects sleep and daily activities.
- 10: Intense pain, severely affects sleep and other daily activities.

(2) *Comfort evaluation:* The BCS is used to evaluate patient comfort at T0 to T8 time points.^[12]^ Lower scores indicate poorer comfort levels.

- 0: Persistent pain.
- 1: No pain at rest, severe pain with deep breathing or coughing.
- 2: No pain at rest, mild pain with deep breathing or coughing.
- 3: No pain with deep breathing.
- 4: No pain with coughing.

(3) *Survival time and antitumor treatment:* Record the postoperative survival time and active antitumor treatment status of patients.

(4) *Opioid adverse reactions and surgical-related indicators:* Record preoperative and postoperative adverse reactions such as constipation, nausea, and vomiting, respiratory depression, as well as sleep duration, diet status, surgical complications. Also record the number of hospital readmissions due to equipment failure or other adverse events.

### 2.6. Statistical analysis

SPSS 19.0 software was used for data analysis. All count data were expressed as number of cases, while measurement data were expressed as mean ± standard deviation (*x* ± SEM). *P* < .05 was considered statistically significant.

## 3. Results

### 3.1. General information

A total of 49 patients were included, with an average age of 68.85 ± 9.89 years. There were 24 male and 25 female patients. All patients had a confirmed diagnosis of primary malignancy, and at admission, they had multiple metastatic lesions throughout the body, losing the opportunity for surgery. After standardized antitumor treatment from the Department of Oncology, they presented with moderate to severe pain. Among them, 19 patients had metastatic lung cancer, 8 had rectal cancer, 8 had liver cancer, 6 had pancreatic cancer, 4 had cervical cancer, 3 had stomach cancer, and 1 had bladder cancer. The diagnostic criteria for refractory cancer pain were strictly met: 1 week after the WHO standardized “3-step analgesic ladder” treatment from the Department of Pain or Oncology, the NRS score was 7.84 ± 0.84, BCS score was 1.12 ± 0.85, or the side effects of analgesic drugs were intolerable (Table 1).

**Table 1:** Comparison of general information (n = 49; *x* ± SEM).

Male/female (n)	Age (yr)	NRS score	BCS score	Multiple organ metastasis primary disease (n)					
				Lung cancer	Colorectal cancer	Cancer of the liver	Pancreatic cancer	Cervical cancer	Else
24/25	68.85 ± 9.89	7.84 ± 0.84	1.12 ± 0.85	19	8	8	6	4	4

### 3.2. NRS and BCS scores at T0 to T8

Compared to T0, the NRS scores at T1 to T8 were significantly lower (*P* < .001), indicating significant improvement in pain intensity. The BCS scores at T1 to T8 were significantly higher than T0 (*P* < .05), indicating improved comfort levels. There were no significant differences in NRS or BCS scores between T1 and T8 (*P* > .05) (Table 2).

**Table 2:** NRS and BCS scores at each time point (n* = *49; *x ± *SEM).

	NRS score	BSC score	Survival rates (%)
T0 (49)	7.84 ± 0.83	1.12 ± 0.86	100% (49/49)
T1 (49)	2.16 ± 0.83# (*P* = .000#)	3.14 ± 0.58# (*P* = .000#)	100% (49/49)
T2 (45)	2.58 ± 1.44#□ (*P* = .000#, *P* = .093□)	3.04 ± 0.64#□ (*P* = .000#, *P* = .421□)	91.83% (45/49)
T3 (36)	2.36 ± 1.37#□ (*P* = .000#, *P* = .313□)	3.14 ± 0.72#□ (*P* = .000#, *P* = .975□)	73.46% (36/49)
T4 (30)	2.33 ± 1.32#□ (*P* = .000#, *P* = .343□)	3.13 ± 0.68#□ (*P* = .000#, *P* = .932□)	61.22% (30/49)
T5 (21)	2.43 ± 0.68#□ (*P* = .000#, *P* = .066□)	3.05 ± 0.67#□ (*P* = .000#, *P* = .343□)	42.85% (21/49)
T6 (17)	2.29 ± 0.77#□ (*P* = .000#, *P* = .326□)	2.94 ± 0.66#□ (*P* = .000#, *P* = .063□)	34.69% (17/49)
T7 (15)	2.27 ± 0.70#□ (*P* = .000#, *P* = .447□)	3.07 ± 0.45#□ (*P* = .000#, *P* = .429□)	30.61% (15/49)
300–400 day			12.24% (6/49)
T8(49)	2.45 ± 0.61#□ (*P* = .000#, *P* = .056□)	3.02 ± 0.56#□ (*P* = .000#, *P* = .261□)	-

Compared with T0.

#*P* < .001; compared with T1.

□ *P* > .05.

The day on admission (T0), the day of discharge (T1), 30 d post-discharge (T2), 60 d post-discharge (T3), 90 d post-discharge (T4), 120 d post-discharge (T5), 150 d post-discharge (T6), 180 d post-discharge (T7), and the day before death (T8).

### 3.3. Comparison of opioid-related adverse reactions and sleep duration

Compared to T0, the number of patients requiring medication for constipation at each time point from T1 to T8 was significantly reduced. The number of patients with improved diet increased significantly, and the duration of high-quality sleep was significantly prolonged. There were no incidents of respiratory depression reported (Table 3).

**Table 3:** Adverse reactions of opioid drugs, sleep time, etc (n* =* 49).

	Constipation (n)	Nausea and vomiting (n)	Respiratory depression (n)	Sleep (n)		
	Need medical treatment	Unable to eat normally		≤1 h/night	1 to 3 h/night	≥4 h/night
T_0_ (49)	34	47	0	37	13	0
T_1_ (49)	16	4	0	0	4	45
T_2_ (43)	9	6	0	0	3	40
T_3_ (36)	10	6	0	0	6	30
T_4_ (30)	7	7	0	0	4	24
T_5_ (21)	11	7	0	0	3	18
T_6_ (17)	6	2	0	0	4	11
T_7_ (15)	7	2	0	0	5	10
T_8_ (49)	–	49	-	17	32	0

The day on admission (T0), the day of discharge (T1), 30 d post-discharge (T2), 60 d post-discharge (T3), 90 d post-discharge (T4), 120 d post-discharge (T5), 150 d post-discharge (T6), 180 d post-discharge (T7), and the day before death (T8).

### 3.4. Postoperative complications, survival time, and adverse event management

The average survival time after IDDS surgery was 135.51 ± 102.69 days. Among the 49 patients, 11 received active antitumor treatment, accounting for 22.44%. Postoperatively, 1 patient had CSF leakage, 3 patients had infections at the butterfly needle insertion site. patients conducted self-evaluation on the information platform according to their actual situation, and doctors provided timely assessment based on the self-evaluation content, a total of 79 adverse events were managed, including 73 patients or their families were actively guided to carry out preliminary treatment and 6 requiring hospital readmission due to catheter blockage (Table 4).

**Table 4:** Postoperative complications of IDDS (n* =* 49).

Postoperative complications (n)		Information platform successfully processed (times)	Return to Hospital (times)	IDDS postoperative	
Cerebrospinal fluid leakage	Wound infection			Continue to antineoplastic therapy (n)	Survival time (d)
1	3	73	6	11 (22.44%)	135.51 ± 102.69

### 3.5. Management of CSF leak

**Figure 1.**
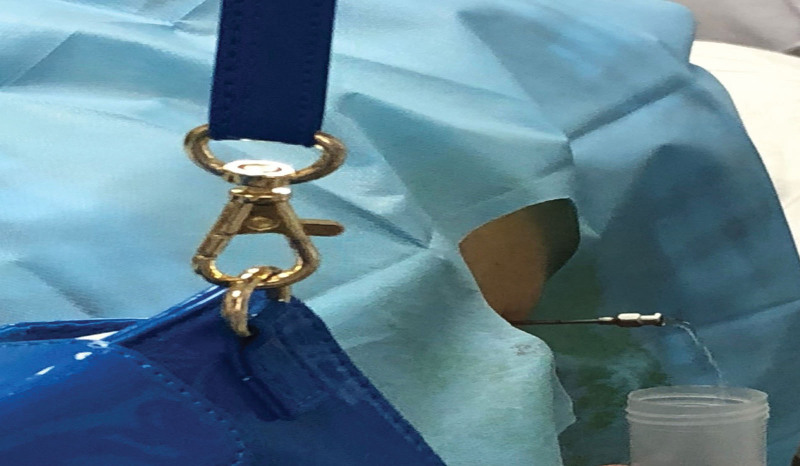
Subarachnoid puncture.

**Figure 2.**
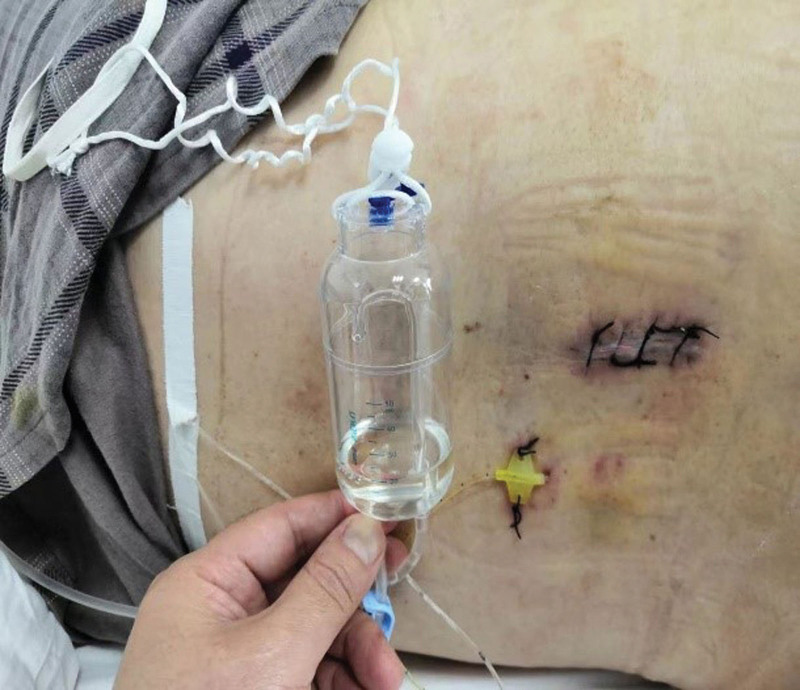
Lumbar cistern cerebrospinal fluid drainage.

**Figure 3.**
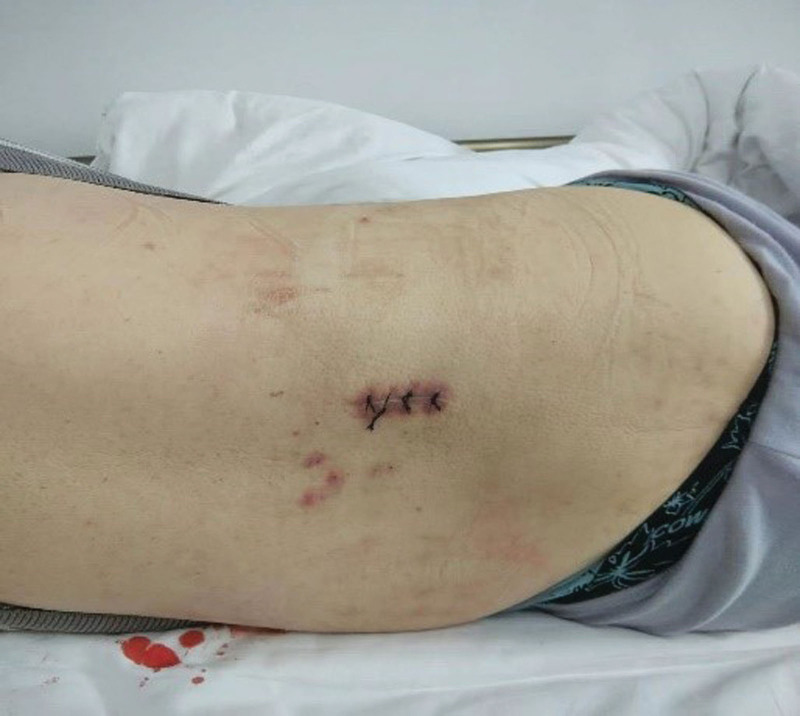
Wound healing after cerebrospinal fluid drainage.

The patient was positioned at the L2 to L3 interspace for drainage of CSF from the lumbar cistern. A lateral approach was used with local anesthesia administered with 3ml of 2% lidocaine. A subarachnoid puncture was performed to visualize the CSF leak (Fig. 1), and a catheter was inserted to the T10 to T11 plane and fixed to the skin. The catheter was connected to a drainage bottle, and CSF was slowly drained at a rate of 70 to 100 mL per day (Fig. 2). The patient remained asymptomatic, without headache or vomiting. After 7 days, the wound had healed, and the drainage catheter was removed (Fig. 3).

## 4. Discussion

“Home analgesia” significantly improves the quality of survival of end-stage cancer patients. But the doctors are difficult to dynamically monitor the pain condition of the patients under “home analgesia” mode. The cancer pain information platform helps the doctors to timely deal with emergencies in the process of analgesia under home analgesia. The doctors can timely assess the pain and analyze the causes, then guide the patients and their families to adjust the morphine dosage and deal with other situations according to the actual situation. On the other hand, the platform reduces the times that the patients go back and forth to the hospital, and significantly improve the quality of survival of end-stage patients. The findings in this study demonstrated that the cancer pain information platform in the home-based analgesia mode yielded favorable outcomes in patients with refractory cancer pain, leading to a considerable enhancement in their quality of life. Simultaneously, the study also discovered that utilizing IT for analgesia at home can decrease aspects such as the risk of infection and the complexity of managing PCA pumps. This suggests that the IT analgesic option under “home analgesia” mode has significant potential for practical use.

Due to cultural, economic, and regional healthcare conditions, most cancer patients tend to live at home during breaks in antitumor treatment, especially for those with advanced cancer pain. Cancer pain is a common and feared symptom among cancer patients. The mechanism of cancer pain is complex. In addition to aggressive antitumor treatment, strong opioid drugs are the main treatment for cancer pain. Even after standardized “3-step analgesic ladder” analgesic treatment by the WHO,^[13–15]^ about 20% of patients still have pain that is difficult to control with medication, or cannot tolerate the side effects of analgesic drugs, which makes it impossible for them to eat normally, sleep well, and reduce social interactions, leading them to abandon further treatment. The data in Table 1 show that 49 patients with advanced cancer pain, with an expected survival time of <3 months, had a NRS score of 7.84 ± 0.84 and a BCS score of 1.12 ± 0.85 after standardized antitumor and analgesic treatment. This seriously reduced the quality of life of these patients. How to improve the quality of life of patients with refractory cancer pain under the “home analgesia” model is currently a hot topic for clinical research that requires multidisciplinary collaboration to solve.

As one of the irreplaceable methods for the treatment of refractory cancer pain in the “4th step,” semi-implanted IDDS has a strong analgesic effect by directly delivering opioids into the subarachnoid space to bind with spinal and supraspinal opioid receptors.^[16]^ It achieves optimal pain intensity control with the smallest dose of opioids, while reducing opioid-related adverse reactions, laying the foundation for the “home analgesia” model.^[17]^ When comparing completely implanted IDDS with semi-implanted IDDS, the latter can be a more favorable option for refractory cancer patients facing economic challenges, as it is more cost-effective. Nevertheless, it encounters elevated susceptibility to infection, challenges in assessing pain intensity, and suboptimal patient adherence. Moreover, the prevailing agreement regarding the identification and management of stubborn cancer pain indicates that it is appropriate for individuals with a projected lifespan of fewer than 3 months, hence restricting the extensive implementation of this method. Facilitating effective communication among patients, family members, and clinicians on PCA pump care and drug replacement further mitigates the widespread adoption of this technology.^[18]^

In the IDDS surgery, analgesic drugs are directly injected into the intrathecal space, where they act on the corresponding sites of the spinal cord to block the transmission of pain signals to the brain, thereby controlling pain. This device is divided into total and semi-implantable intrathecal infusion systems. In the study, the combined use of intrathecal opioids and local anesthetics for acute pain can prolong analgesic duration, reduce dosage and adverse reactions.^[19]^ The results showed a 3-month survival rate of 61.22% with a mean survival time of 135.51 ± 102.69 days, significantly longer than the expected 90-day survival period. After IDDS surgery, 11 patients continued to receive routine and standardized antitumor treatment, effectively controlling tumor cell proliferation. This suggests a significant reduction in opioid-related adverse reactions, a significant decrease in postoperative pain intensity, and stable pain control. The patients’ sleep quality, diet, and immune function improved, leading to an improvement in their quality of life.

However, CFS leakage is a potential complication. Persistent CFS leakage can affect patients’ daily lives, increasing the risk of complications and death. Therefore, perioperative prevention is the focus of CFS leakage management. CFS leakage refers to the phenomenon where CFS leaks from the cranial defect covered by the dura mater through the nasal cavity, external auditory canal, or open wound. Once CFS leakage occurs, it should be diagnosed early and accurately, and appropriate treatment methods should be selected to improve the patient’s prognosis.^[20]^

Infection refers to localized skin puncture site infections in this study, which are related to multiple factors, such as the patient’s underlying condition, the care of the insertion site of butterfly needle, and the length of time the patient has been alive. In this study, 1 patient had an incision that did not heal due to CFS leakage, which may be related to loosening of the catheter fixation sutures and early postoperative activity. Fortunately, the CFS leakage was properly managed through continuous lumbar epidural drainage and wound dressing changes for 7 days. Additionally, 3 cases of infection at the insertion site of the butterfly needle were appropriately treated and controlled. This indicates that implantation of the semi-implantable IDDS under strict aseptic conditions is safe and feasible.

In the “home analgesia” model, timely treatment of mechanical failures, dynamic monitoring of pain intensity, and timely replacement of analgesic doses based on changes in pain intensity are common issues. Hui-jing et al^[21]^ applied information systems to promptly identify and rapidly address analgesic issues in hospitalized cancer patients; Some researchers^[21–23]^ developed an information management platform that proactively monitors pain intensity in patients receiving home analgesia, providing consultation, online consultation, and training services. This study conducted a standardized training program for medical staff, patients, and family members on pain intensity assessment, information system operation, and common troubleshooting, enabling them to fully participate in pain self-management. With the help of this platform, medical staff can dynamically monitor patients’ pain intensity and guide patients to adjust opioid dosages. This study showed that during home analgesia, the information platform can effectively guide patients or their families to address problems in a short time, avoiding the need for hospital visits and reducing patient burden. Only 6 people returned to the hospital for connection line blockage treatment, with no cases of respiratory depression. This fully confirms that cancer pain information construction can fully involve patients in standardized pain management, enabling timely and effective treatment of analgesic issues.

The reasons for not using internal pumps in this study are as follows: first, Zunyi City is a third or fourth-tier city with a per capita disposable income: (2022) 25,000 yuan/per year for urban residents and 14,000 yuan/per year for rural residents. The fully implantable morphine pump is an imported material that costs around 150,000 RMB and is fully self-funded. The cost of antitreatment for cancer patients far exceeds the entire income of the whole family, and patients cannot afford such high costs. Second, the products used in this study are domestic products with low cost, only 1/10 of the cost of fully implanted morphine pumps, and are reimbursed by Guizhou Provincial Health Insurance. The materials greatly reduce the economic burden of patients. Third, the patient’s condition has reached advanced stage, and have lost the opportunity to treat the tumor primary disease. The survival time have been assessed to be about 1 to 6 months. The patients put only analgesia for the main purpose of treatment. Because the conventional analgesic treatment (oral opioid drugs) has not been able to meet the demand for analgesia, so the simple morphine pump has become a necessary means of choice.

### 4.1. Limitations

This study requires long-term tracking and observation, with a long research cycle, resulting in high research costs. At the same time, the research results may be affected by time factors. A large sample size is still required to avoid inaccuracy and bias in the research results.

## 5. Conclusion

In summary, the semi-implanted IDDS combined with the cancer pain information platform can be applied to the “home analgesia” model to standardize the management and effectively control refractory cancer pain, prolong high-quality sleep time, reduce opioid side effects, improve diet, and significantly improve the quality of life of patients with refractory cancer pain under the “home analgesia” model. This method is safe and cost-effective, and is suitable for patients with an expected survival time of ≥3 months. It is worth promoting. Actively promoting community medical staff to participate in cancer pain management and providing on-site services will further improve patients’ quality of life. Whether it can prolong patients’ survival time still needs to be further confirmed by more research data.

## Author contributions

**Conceptualization:** Yongyong Ding, Hong Deng.

**Data curation:** Yongyong Ding, Hong Deng, Jie Peng.

**Investigation:** Yongyong Ding, Hong Deng, Jie Peng.

**Methodology:** Yongyong Ding, Jie Peng.

**Project administration:** Yongyong Ding, Hong Deng.

**Resources:** Yongyong Ding, Jie Peng.

**Validation:** Yongyong Ding, Jie Peng.

**Writing – original draft:** Yongyong Ding, Hong Deng.

**Writing – review & editing:** Yongyong Ding, Hong Deng, Jie Peng.

**Formal analysis:** Hong Deng.

**Supervision:** Hong Deng.

**Visualization:** Hong Deng, Jie Peng.

## References

[1] Mestdagh F Steyaert A Lavand’homme P. Cancer pain management: a narrative review of current concepts, strategies, and techniques. Curr Oncol. 2023;30:6838–58. 37504360 10.3390/curroncol30070500 PMC10378332

[2] Forte AJ Guliyeva G McLeod H. The impact of optimism on cancer-related and postsurgical cancer pain: a systematic review. J Pain Symptom Manage. 2022;63:e203–11. 34563629 10.1016/j.jpainsymman.2021.09.008

[3] Mercadante S. Refractory cancer pain and intrathecal therapy: critical review of a systematic review. Pain Ther. 2023;12:645–54. 37055698 10.1007/s40122-023-00507-z PMC10199986

[4] Hochberg U Ingelmo P Solé E. Early interventional treatments for patients with cancer pain: a narrative review. J Pain Res. 2023;16:1663–71. 37223437 10.2147/JPR.S405808 PMC10202202

[5] De Andres J Hayek S Perruchoud C. Intrathecal drug delivery: advances and applications in the management of chronic pain patient [published correction appears in Front Pain Res (Lausanne). 2023 Apr 11;4:1190014]. Front Pain Res (Lausanne). 2022;3:900566. 35782225 10.3389/fpain.2022.900566 PMC9246706

[6] Ma Y Deng Z Feng X. Effects of hydromorphone-based intravenous patient-controlled analgesia with and without a low basal infusion on postoperative hypoxaemia: study protocol for a randomised controlled clinical trial. BMJ Open. 2022;12:e064581. 10.1136/bmjopen-2022-064581 PMC9670915 36385038

[7] Peng M Chungrui L Qian Z. Management of severe cancer pain by intrathecal morphine infusion via subcutaneous port. Chin J Pain Med. 2016;22:5–8.

[8] Schultz DM Orhurhu V Khan F. Patient satisfaction following intrathecal targeted drug delivery for benign chronic pain: results of a single-center survey study. Neuromodulation. 2020;23:1009–17. 32378289 10.1111/ner.13167 PMC7687151

[9] Rafii F Taleghani F Khatooni M. Barriers to effective cancer pain management in home setting: a qualitative study. Pain Manag Nurs. 2021;22:531–8. 33323346 10.1016/j.pmn.2020.11.003

[10] Su Q Wang H Fan L. The impact of home and community care services pilot program on healthy aging: a difference-in-difference with propensity score matching analysis from China. Arch Gerontol Geriatr. 2023;110:104970. 36842402 10.1016/j.archger.2023.104970

[11] Geerling JI van der Linden YM Raijmakers NJH. Randomized controlled study of pain education in patients receiving radiotherapy for painful bone metastases. Radiother Oncol. 2023;185:109687. 37169300 10.1016/j.radonc.2023.109687

[12] Chen S Guo Z Wei X. Efficacy of preemptive intercostal nerve block on recovery in patients undergoing video-assisted thoracic lobectomy. J Cardiothorac Surg. 2023;18:168. 37118846 10.1186/s13019-023-02243-z PMC10148478

[13] Mercadante S. Managing difficult pain conditions in the cancer patient. Curr Pain Headache Rep. 2014;18:395–7. 24407750 10.1007/s11916-013-0395-y

[14] Diaz Arguello OA Haisma HJ. Apoptosis-inducing TNF superfamily ligands for cancer therapy. Cancers (Basel). 2021;13:1543. 33801589 10.3390/cancers13071543 PMC8036978

[15] ALMouaalamy N Alharbi ZM Aldosari FM. The practice of pain assessment and management in a tertiary oncology center. Cureus. 2021;13:e18837. 34804692 10.7759/cureus.18837 PMC8594563

[16] Dupoiron D Duarte R Carvajal G. Rationale and recent advances in targeted drug delivery for cancer pain: is it time to change the paradigm? Pain Physician. 2022;25:E414–25. 35652767

[17] Duarte R Copley S Nevitt S. Effectiveness and safety of intrathecal drug delivery systems for the management of cancer pain: a systematic review and meta-analysis. Neuromodulation. 2023;26:1126–41. 35422368 10.1016/j.neurom.2022.03.003

[18] Goel V Yang Y Kanwar S. Adverse events and complications associated with intrathecal drug delivery systems: insights from the Manufacturer and User Facility Device Experience (MAUDE) Database. Neuromodulation. 2021;24:1181–9. 33306248 10.1111/ner.13325 PMC9034464

[19] Capozza MA Triarico S Mastrangelo S. Narrative review of intrathecal drug delivery (IDD): indications, devices and potential complications. Ann Transl Med. 2021;9:186. 33569488 10.21037/atm-20-3814 PMC7867880

[20] Tang J Lu Q Li Y. Risk factors and management strategies for cerebrospinal fluid leakage following lumbar posterior surgery. BMC Surg. 2022;22:30. 35090413 10.1186/s12893-021-01442-6 PMC8800267

[21] Hui-jing Z Xin-jun W Sheng L. Clinical observation of self-controlled analgesia of wireless analgesia pump system for cancerous outbreak pain. J Clin Expmed. 2020;11:1125–8.

[22] Weiwei H Feng J Hongwei H. Clinical application of intelligent pain management system for cancer pain control. Chin J Pain Med. 2015;21:107–10.

[23] Yinghao W Xiaoqing S Juan T. Construction of cancer pain whole-process management system based on BYOD technology. Contemporary Med. 2017;23:3.

